# Genetic Case-Control Study for Eight Polymorphisms Associated with Rheumatoid Arthritis

**DOI:** 10.1371/journal.pone.0131960

**Published:** 2015-07-06

**Authors:** Mohamed N. Saad, Mai S. Mabrouk, Ayman M. Eldeib, Olfat G. Shaker

**Affiliations:** 1 Biomedical Engineering Department, Minia University, Minia, Egypt; 2 Biomedical Engineering Department, Misr University for Science and Technology, 6th of October City, Egypt; 3 Systems and Biomedical Engineering Department, Faculty of Engineering, Cairo University, Giza, Egypt; 4 Medical Biochemistry and Molecular Biology Department, Faculty of Medicine, Cairo University, Giza, Egypt; IIBB-CSIC-IDIBAPS, SPAIN

## Abstract

Rheumatoid arthritis (RA) is an autoimmune disease which has a significant socio-economic impact. The aim of the current study was to investigate eight candidate RA susceptibility loci to identify the associated variants in Egyptian population. Eight single nucleotide polymorphisms (SNPs) (*MTHFR*—C677T and A1298C, *TGFβ1* T869C, *TNFB* A252G, and *VDR*—ApaI, BsmI, FokI, and TaqI) were tested by genotyping patients with RA (n = 105) and unrelated controls (n = 80). Associations were tested using multiplicative, dominant, recessive, and co-dominant models. Also, the linkage disequilibrium (LD) between the *VDR* SNPs was measured to detect any indirect association. By comparing RA patients with controls (*TNFB*, BsmI, and TaqI), SNPs were associated with RA using all models. *MTHFR* C677T was associated with RA using all models except the recessive model. *TGFβ1* and *MTHFR* A1298C were associated with RA using the dominant and the co-dominant models. The recessive model represented the association for ApaI variant. There were no significant differences for FokI and the presence of RA disease by the used models examination. For LD results, There was a high D′ value between BsmI and FokI (D′ = 0.91), but the r^2^ value between them was poor. All the studied SNPs may contribute to the susceptibility of RA disease in Egyptian population except for FokI SNP.

## Introduction

Rheumatoid arthritis (RA) is an autoimmune disease which is considered genetically complex. RA is the leading cause of bone loss and chronic inflammation of the joints, most prominently in white populations. The prevalence of the disease in women is twice that in men. RA attacks the body most often at the age of 40. Over the past 40 years, statistical geneticists have facilitated the discovery of RA biomarkers. Multiple methods have been developed to detect the association between the examined SNPs and disease susceptibility. Although *HLA-DRB1* was considered the most widely studied and the most associated gene with RA susceptibility, SNPs at other gene loci may contribute to the disease. The genetic etiology of the disease is still an open question [1]. The examined genes in our study were selected for their critical role in immunogenetics and the contradictory results of their SNPs in the association with RA susceptibility in different populations.

The most popular statistical models for measuring the association between a genotype and a phenotype are multiplicative, dominant, recessive, and co-dominant models. These models differ in the identification of the exposed group and the unexposed group in cases and controls as shown in Table 1. For the multiplicative model, analysis should be done using alleles instead of genotypes [2, 3].

**Table 1:** The Exposed Group and the Unexposed Group in the Four Used Models.

Model	Exposed	Unexposed
Multiplicative	Major Allele	Minor Allele
Dominant	Major Homozygote	Minor Homozygote & Heterozygote
Recessive	Major Homozygote & Heterozygote	Minor Homozygote
Co-dominant	Major Homozygote	Minor Homozygote or Heterozygote

The co-dominant model is the only one comparing the three categorical genotypes without assuming any relationship between disease and genotypes. So, the co-dominant model has two degrees of freedom (DF). All other models have one DF. The one DF tests are more popular than the two DF tests because of the simplicity of the one DF tests implementation through the 2x2 contingency tables. Also, the one DF tests have higher statistical power than the two DF tests [4].

In our study, the examined eight SNPs are located within four genes which are methylene tetrahydrofolate reductase (*MTHFR*), transforming growth factor beta (*TGFβ1*), tumor necrosis factor beta (*TNFB*), and vitamin D receptor (*VDR*). The C677T and A1298C are common polymorphisms in the *MTHFR* gene [5]. The *MTHFR* gene is a candidate biomarker for RA susceptibility. Homocysteine has been recorded at high levels in RA patients, which is related to the *MTHFR* gene [6]. The allele (677T) has been found to exhibit lower MTHFR enzyme activity and has been implicated in average elevated levels of homocysteine [7]. The 1298C (minor) allele has a lower effect on MTHFR enzyme than the 677T [8, 9].

The T869C is a common polymorphism within the *TGFβ1* gene [10]. The *TGFβ1* gene is a strong candidate biomarker for RA susceptibility. The TGFβ1 protein has been found in the synovial fluid of RA patients [11]. The T869C polymorphism is associated with the soluble TGFβ1 serum levels [12].

The A252G polymorphism is located at position 1069 of intron 1 of the *TNFB* gene [13]. The TNFB is considered as proinflammatory immunostimulatory cytokine. The TNFB cytokine has been detected in nine RA patients (four synovial fluid /serum pairs, three synovial fluid and two sera) out of 27 examined RA patients [14]. The A252G polymorphism influences adhesion molecules and cytokines from different types of leukocytes [15].

ApaI, BsmI, FokI, and TaqI are common polymorphisms within the *VDR* gene [16]. The VDR protein, through the vitamin D endocrine system, has been implicated in the metabolic pathways involved in the immune response. It plays an important role in absorption of calcium, promoting monocyte differentiation, inhibiting lymphocyte proliferation and secretion of cytokines, such as interleukin (IL)-2, interferon gamma (IFNγ), and IL-12 [17, 18].

There are two objectives of our present case-control study. The first objective was to study the direct association between each studied SNP and RA susceptibility. The second objective was to measure the linkage among the four *VDR* SNPs and/or the two *MTHFR* SNPs to detect any indirect association in case of no direct association. The studied parameters are linkage disequilibrium (LD) and odds ratio (OR). These two parameters could be measured using case-control samples.

## Patients and Methods

### Ethics Statement

The study was approved by the Ethical Committee of Faculty of Medicine, Cairo University, and an oral and written informed consent was obtained from all participants.

### Patients

In total, 185 subjects were enrolled in the case-control study: 105 RA patients (89 women and 16 men) and 80 unrelated ethnically matched healthy controls (69 women and 11 men). All subjects in our analysis were Egyptians and were recruited from Rheumatology Department and Outpatient Clinics of Cairo University Hospitals (Kasr El-Aini hospital). RA patients were diagnosed by physician investigators and followed the 1987 American College of Rheumatology (ACR) criteria [19]. DAS28 (Disease Activity Score in 28 Joints), which is a validated score for established RA, was used as a measure for disease activity. No signs of RA, such as joint stiffness in the morning, positive rheumatoid factor (RF) and citrulline antibody or the findings of rheumatoid nodules were observed in controls. Patients with other autoimmune diseases or inflammatory disorders unrelated to RA were not included.

### Molecular Genetic Methods

DNA was extracted from peripheral blood using a QIAamp DNA Blood Mini Kit (Qiagen, Valencia, CA, USA) according to the manufacturer's protocol to be used for genotyping of the eight SNPs *MTHFR* C677T, *MTHFR* A1298C, *TGFβ1* T869C, *TNFB* A252G, ApaI, BsmI, FokI, and TaqI.

#### *MTHFR* C677T genotyping

One set of forward 5'-CAT CCC TAT TGG CAG GTT AC-3' and reverse 5'-GAC GGT GCG GTG AGA GTG- 3' primers were used for the amplification of a fragment of 265 bp, and then the amplified fragments were digested with the HinfI enzyme. The PCR profile was: initial denaturation at 95°C for 5 min, denaturation at 94°C for 30 sec, annealing at 59°C for 30 sec, extension at 72°C for 30 sec for 35 cycles and followed at 72°C for 10 min. At position 677 (rs1801133) of the *MTHFR* gene, the C wild base, replaced by the T base, produces a cut site for the HinfI enzyme, which cuts the amplicons into two fragments of 171 and 94 bp. Then, the CC genotype would be reflected by a single band of 265 bp (uncut), the CT genotype by three bands of 265, 171 and 94 bp, and the TT genotypes by two bands of 171 and 94 bp.

#### *MTHFR* A1298C genotyping

One set of forward 5'-CTT TGG GGA GCT GAA GGA CTA CTA C-3' and reverse 5'-CAC TTT GTG ACC ATT CCG GTT TG-3' primers was used for the amplification of a fragment of 241 bp and then the amplified fragment was digested with the MboII enzyme. The PCR profile was: initial denaturation at 95°C for 5 min, denaturation at 94°C for 30 sec, annealing at 51°C for 30 sec, extension at 72°C for 30 sec for 35 cycles and followed at 72°C for 10 min. At position 1298 (rs1801131) of the *MTHFR* gene, the transversion of the wild A base, to C base produces a cut site for the MboII enzyme, which cuts the PCR product into two fragments of 211 and 30 bp. Then, the AA genotype results in a single band of 241 bp (uncut), the AC genotype produces three bands of 241, 211 and 30 bp, and the CC genotype produces two bands of 211 and 30 bp. The digestion of 10 μl of PCR products was carried out with 1.5 U of the MboII restriction enzyme in 37°C for two hours.

#### *TGFβ1* T869C genotyping

DNA was genotyped by specific primers: 5'-TTCCCTCGAGGCCCTCCTA-3' and 5'-GCCGCAGCTTGGACAGGATC-3' to amplify a fragment of the *TGFβ1* gene (rs1982073), with denaturation at 96°C for 10 min, followed by 35 cycles at 96°C for 75 sec, 62°C for 75 sec, 73°C for 75 sec, and a final extension at 73°C for five min. MspA1I (New England Biolabs, Hitchin, UK) digestion of the 294 bp fragments at 37°C for 3 hours resulted in fragments of the T allele of 161, 67, 40, and 26 bp, and the C allele of 149, 67, 40, 26, and 12 bp. The samples were then analyzed by electrophoresis on 4% agarose gel stained with ethidium bromide and the genotypes were determined.

#### *TNFB* A252G genotyping

Genotypes for *TNFB* (rs909253) were determined by polymerase chain reaction-restriction fragment length polymorphism (PCR-RFLP). Specific oligonucleotide primers were used: 5'-CCGTGCTTCGTGCTTTGGACTA-3' and 5'-AGAGGGGTGGATGCTTGGGTTC-3', 782 bp fragments were amplified for the first intron of the *TNFB* gene. PCR products were digested with the *Nco*I restriction enzyme and analyzed on 2% agarose gel. The *TNFB* digested product generated fragments of 586 and 196 bp or 782 bp for TNFB*1 or TNFB*2 homozygous individuals, respectively. For heterozygous individuals, three fragments (196, 586 and 782 bp) are detected.

#### *VDR* (ApaI, BsmI, FokI, and TaqI) genotyping

The *VDR* ApaI (rs7975232), the BsmI (rs1544410), the FokI (rs2228570), and the TaqI (rs731236) SNPs were detected by PCR–RFLP according to the manufacturer’s instructions (New England BioLabs, Ipswich, USA). DNA digested fragments were separated in 3% agarose gels and visualized by ethidium bromide staining. Primer sequences and conditions for PCR–RFLP analyses were presented in S1 Table, as described earlier [20].

### Materials

The marker checks, the normalized D (D′), and the correlation between pair of SNPs (r^2^) were measured using *Haploview 4*.*2* (from Daly Lab at the Broad Institute, Cambridge, MA 02141, USA) [21]. The odds ratio (OR), its confidence interval (CI), and Pearson chi square (χ^2^) test for goodness of fit were measured using *SNPAnalyzer 2*.*0* (from Bioinformatics Unit, ISTECH Inc., Republic of Korea) [22, 23]. The statistical power was measured using *Genetic Power Calculator* (from the Purcell lab, http://pngu.mgh.harvard.edu/~purcell/gpc/) [24].

### Statistical Methods

The used bi-allelic marker checks through this study were a) genotype percentage, b) minor allele frequency, c) heterozygosity, and d) Hardy-Weinberg equilibrium P-value. The association between the eight genetic polymorphisms and susceptibility to RA was assessed by the ORs with their corresponding 95% CI under four genetic models including the multiplicative model, the dominant model, the recessive model, and the co-dominant model. A two-sided p-value less than 0.01 was considered statistically significant. The input parameters used in calculating power could be listed as high risk allele frequency, disease prevalence in the general population (1%), genotypic relative risks, type I error rate (0.05), no. of cases (105), and no. of controls (80). Also, the indirect association between any of the eight genetic polymorphisms and susceptibility to RA was detected by D′ and r^2^ which are the most common measures of LD. An indirect association was considered when D′ value was more than or equal 0.8 and r^2^ value was more than or equal 0.8 between a directly associated SNP and an unassociated SNP.

**Fig 1:**
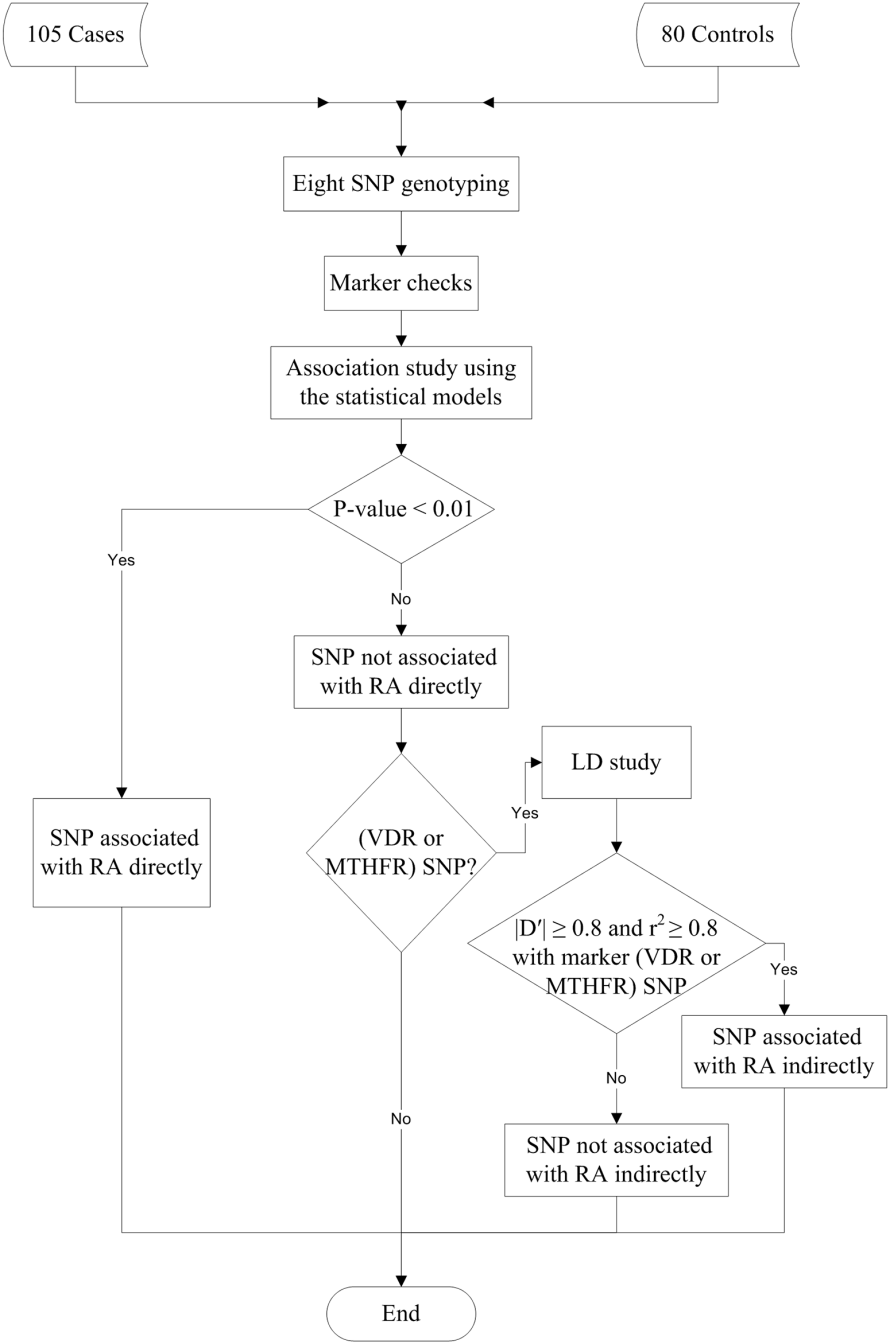
Flow Chart of the Proposed Study. The eight SNPs were genotyped to detect the association with RA susceptibility either directly or indirectly. The SNP that fails to associate with the phenotype directly will undergo an LD study to detect any indirect association as a surrogate for direct disease association.

The flow chart shown in Fig 1 illustrated the proposed association scheme. The LD should be measured between bi-allelic SNPs that lie on the same chromosome. So, LD could be measured between *MTHFR* (C677T and A1298C) SNPs to detect any indirect variant. Also, the *VDR* (ApaI, BsmI, FokI, and TaqI) SNPs were appropriate for LD study as they were all located on chromosome 12.

## Results

Eighty six percent of control group were females. Eighty five percent of RA patients were females. The mean age of RA women ± standard deviation (SD) was 40.84 ± 11.01 years. The mean age of RA men ± SD was 52.25 ± 13.46 years. The average disease duration of RA women ± SD was 6.24 ± 4.17 years. The average disease duration of RA men ± SD was 2.85 ± 4.38 years.

We conducted a check for conformance with HWE, MAF, and the percentage of individual successfully genotyped for each SNP. The minimum accepted genotype percentage was 75%. The markers that were significantly deviated from HWE (HW p-value < 0.005) were excluded. The minimum accepted MAF was 0.01. The results of the marker checks stage are shown in Table 2. All the SNPs for all individuals were fully genotyped and all SNPs passed the marker checks stage. Also, information about each SNP (ID, physical position, chromosome no., major allele, and minor allele) was provided in Table 2. At last, a data set including 1480 SNPs corresponding to 185 uncorrelated individuals was utilized in our study. The power results of the study were shown in S2 Table.

**Table 2:** Marker Checks of the Studied SNPs.

	*MTHFR* C677T	*MTHFR* A1298C	*TGFβ1* 869C	*TNFB* A252G	ApaI	BsmI	FokI	TaqI
**ID**	rs1801133	rs1801131	rs1982073	rs909253	rs7975232	rs1544410	rs2228570	rs731236
**Position**	11856378	11854476	41858921	31540313	48238837	48239835	48272895	48238757
**Chromosome**	1	1	19	6	12	12	12	12
**Alleles Major:Minor**	C:T	A:C	T:C	A:G	G:T	G:A	C:T	T:C
**Genotype %**	100%	100%	100%	100%	100%	100%	100%	100%
**MAF**	0.254	0.416	0.473	0.343	0.341	0.4	0.219	0.395
**ObsHET**	0.378	0.465	0.546	0.459	0.422	0.476	0.362	0.465
**PredHET**	0.379	0.486	0.499	0.451	0.449	0.48	0.342	0.478
**HW P-value**	1	0.6329	0.2667	0.9567	0.4773	0.9934	0.5924	0.8002

**Fig 2:**
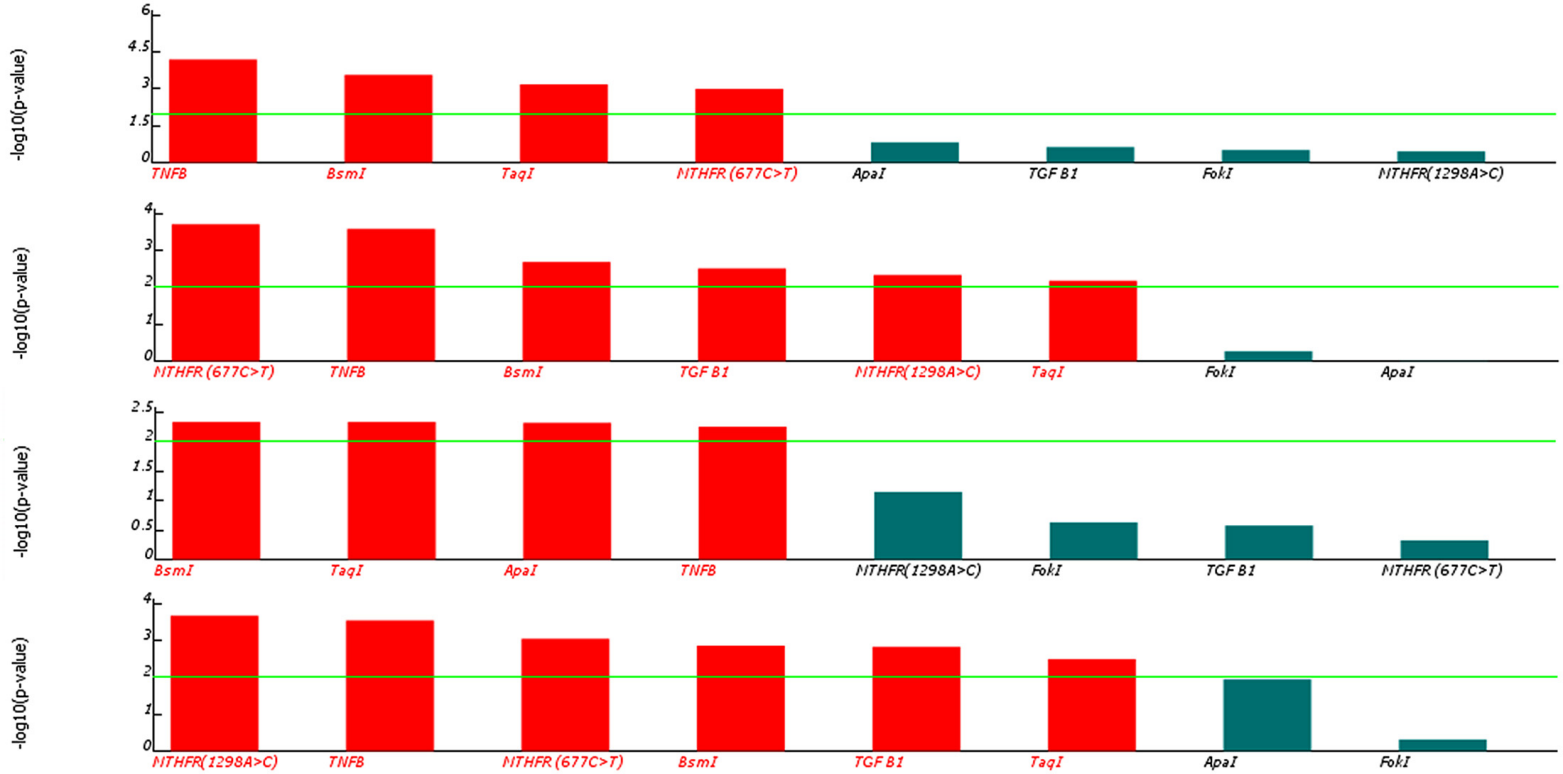
Association Analyses for Examined SNPs with RA Disease. (a) Multiplicative model. (b) Dominant model. (c) Recessive model. (d) Co-dominant model. The horizontal line in each model represents the significance level of the P value (0.01). The figure was generated using the *SNPAnalyzer 2*.*0* program.

Table 3 represented the association between the examined SNPs and RA disease. A graphical representation of the association results for the studied SNPs was shown in Fig 2. The red color in Fig 2 demonstrated a statistically significant SNP. From Table 3 and Fig 2, *TNFB*, BsmI, and TaqI showed significant association with RA susceptibility with the four used models. *MTHFR* C677T expressed significant association with RA susceptibility with all models except the recessive model. *TGFβ1* and *MTHFR* A1298C showed significant association with RA susceptibility with the dominant and co-dominant models. ApaI imposed significant association with RA susceptibility with only the recessive model. FokI did not show any significant association with RA directly with any of the used models.

**Table 3:** Case-Control Study—SNP Analysis.

		*MTHFR C677T*	*MTHFR A1298C*	*TGFβ1 T869C*	*TNFB A252G*	*ApaI*	*BsmI*	*FokI*	*TaqI*
Multiplicative Model	P	0.001	0.348	0.233	6.435E-05	0.151	2.711E-04	0.307	6.588E-04
	χ2	10.825	0.88	1.423	15.97	2.063	13.26	1.044	11.602
	OR (95% CI)	0.433(0.2610.718)	1.221(0.8051.852)	0.778(0.5151.176)	2.424(1.5633.759)	0.725(0.468–1.125)	2.188(1.4313.346)	0.769(0.4641.274)	2.082(1.3613.183)
Dominant Model	P	1.975E-04	0.005	0.003	2.634E-04	0.974	0.002	0.545	0.007
	χ2	13.854	8.017	8.747	13.314	0.001	9.483	0.367	7.356
	OR (95% CI)	0.315(0.1690.584)	2.503(1.3164.763)	0.364(0.1840.72)	3.131(1.6795.838)	0.99(0.5521.777)	2.704(1.4225.139)	0.832(0.4581.51)	2.366(1.2614.437)
Recessive Model	P	0.474	0.072	0.266	0.006	0.005	0.005	0.235	0.005
	χ2	0.513	3.247	1.239	7.668	7.936	8.004	1.411	8.004
	OR (95% CI)	0.638(0.1852.199)	0.482(0.216–1.077)	1.505(0.731–3.102)	3.808(1.405–10.321)	0.224(0.073–0.684)	3.167(1.388–7.226)	0.209(0.025–1.771)	3.167(1.388–7.226)
Co-dominant Model	P	9.054E-04	2.153E-04	0.001	2.895E-04	0.011	0.001	0.483	0.003
	χ2	14.014	16.887	13.015	16.294	8.961	13.157	1.453	11.474

**Fig 3:**
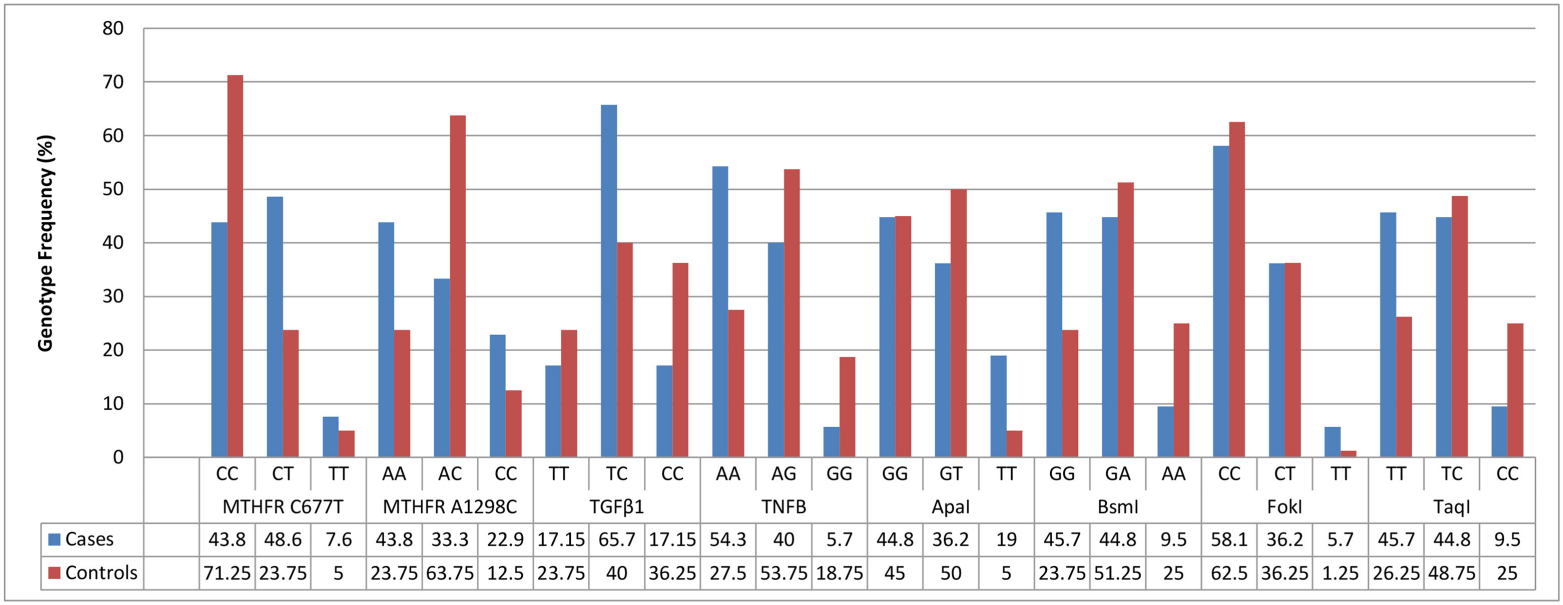
Genotype Distributions in RA Patients and Controls. For the eight SNPs, the genotype frequencies were illustrated as bar charts for cases and controls.

Genotype frequencies for each polymorphism for patients and controls were presented in Fig 3. From Table 3 and Fig 3, the genotypes/alleles that increases or decreases the susceptibility for RA could be addressed. Carriers of *MTHFR* C677T (CC) genotype had a decreased risk of RA. The (CT) genotype and (T) allele of the C677T variant was significantly associated with increased risk of RA. Patients bearing *MTHFR* A1298C (AA) genotype showed a trend to have an increased risk for RA. The (AC) genotype of the A1298C had a protective role against RA. Carriers of *TGFβ1* (TC) genotype had an increased risk of RA. The OR under the dominant model provided an evidence of association for *TGFβ1* with RA showing protective role for the (CC) genotype. Carriers of *TNFB* (AA) genotype and (A) allele had an increased risk of RA whereas individuals harboring the *TNFB* (AG) and (GG) genotypes were refractory to the disease. The (TT) genotype of the ApaI variant was significantly associated with increased risk of RA. The (GG) genotype of the BsmI predisposed its carrier to RA while the (AA) genotype and (A) allele of the BsmI had a protective role against RA. The (TT) genotype and (T) allele of the TaqI biomarker showed statistical influence in RA patients whereas the (CC) genotype of the TaqI seemed to be protective to RA. There was no direct association between FokI polymorphism and RA due to the comparable frequencies between cases and controls.

**Fig 4:**
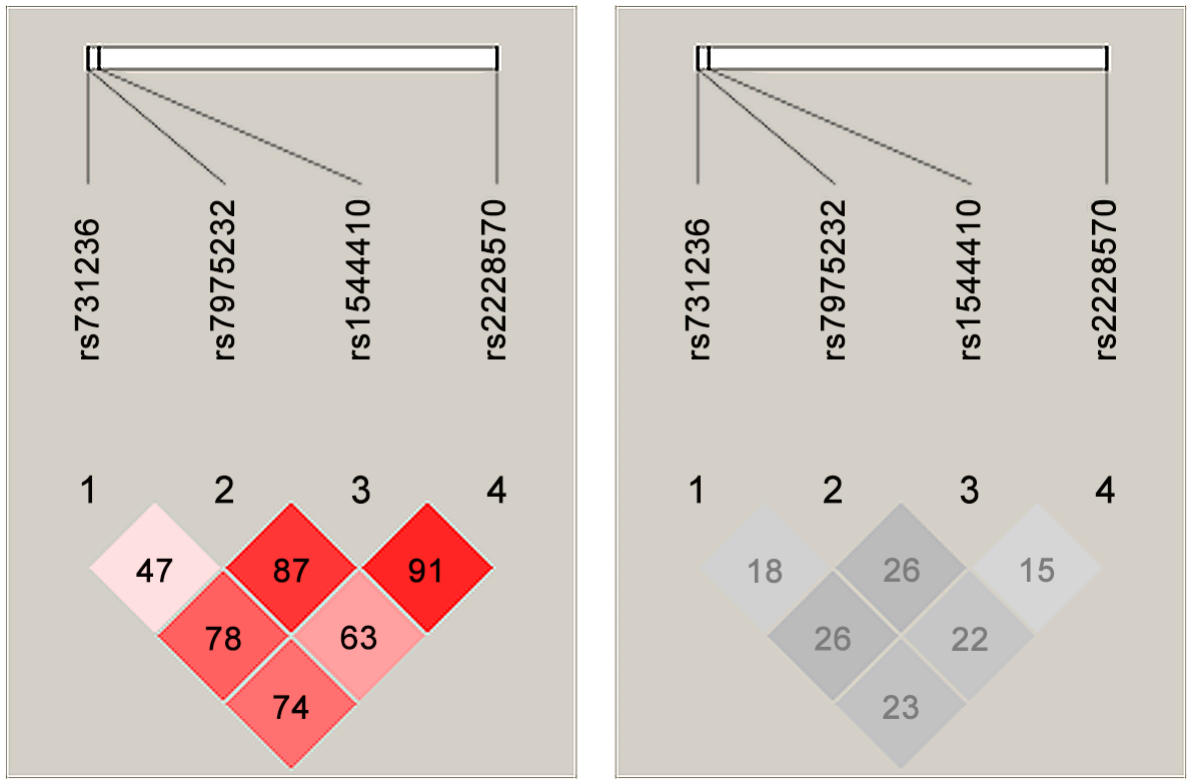
Pairwise LD Plot for the *VDR* SNPs. The ID of each SNP was taken as reference. The rs731236, rs7975232, rs1544410, and rs2228570 correspond to TaqI, ApaI, BsmI, and FokI respectively. (a) D′ values. (b) r^2^ values. The plot was generated using the *Haploview 4*.*2* program.

FokI was the only variant that was not associated with RA susceptibility directly. LD between the *VDR* SNPs was measured to detect whether FokI variant was associated with RA indirectly. LD results for all *VDR* SNPs were shown in Fig 4. The *VDR* SNPs were shown in the order in which they appear on the genome. Each D′ value and r^2^ value in the plot were multiplied by 100. Generally, the nearer SNPs tend to have high D′ values while the SNPs that are farther apart tend to have lower D′ values. The four *VDR* SNPs were in close proximity. Despite these proximities, the LD results were poor. Only two D′ values were greater than 0.8 which were between (FokI, ApaI) and BsmI. All the r^2^ values were poor. While the D′ value between FokI and BsmI equaled 0.91, but the r^2^ value between them equaled 0.15. This was due to one SNP being much rarer than the other. So, the SNPs could not substitute each other. At last, both D′ and r^2^ values must be specified to take the correct decision.

## Discussion

RA is an autoimmune chronic disease that affects body’s joints and bones. RA pathogenesis is an active area of research including several genes. *MTHFR*, *TGFβ1*, *TNFB*, and *VDR* genes have generated great interest in RA pathogenesis [1, 25–27].

In the present study, the distribution of genotypes and alleles of eight SNPs was used to examine the association with RA susceptibility in the Egyptian population (105 RA patients and 80 healthy controls). The examined SNPs belonged to four genes (*MTHFR*, *TGFβ1*, *TNFB*, and *VDR*). Seven SNPs were considered candidates for RA susceptibility which are *TNFB* (rs909253), BsmI (rs1544410), TaqI (rs731236), *MTHFR* C677T (rs1801133), *TGFβ1* (rs1982073), *MTHFR* A1298C (rs1801131), and ApaI (rs7975232). There was no proof of association for FokI (rs2228570) with RA.

The association between RA and the studied polymorphisms has been examined in several studies. Contradictory results had arisen due to different populations, the age of the subjects, and the sample sizes of these studies. The genetic characteristics of the modern Egyptian population are a mixture of European, Middle Eastern, and African populations [28]. This issue could explain the agreement/disagreement of our results with published data of other populations. Table 4 showed the influential genotype/allele in case of the presence of an association for the studied SNP with RA in the corresponding population. The conflicting results in Table 4 might be due to the small sample size of most of the included studies.

**Table 4:** Association Status with RA of Our Study and Previous Studies.

	Population	No. of cases	No. of controls	Genotype	Genotype Frequency (cases—controls)	Allele	Allele Frequency (cases—controls)	Association with RA	Reference
***MTHFR* C677T**	Egyptian (our study)	105	80	CC	46–57			Protective	
				CT	51–19	T	67–27	Susceptible	
	Jewish	93	377					No association	[29]
	Italian	217	251					No association	[5]
	African-American	138	53					No association	[7]
	Caucasian	393	50					No association	[7]
	Turkish	147	150			T	51–32	Susceptible	[30]
***MTHFR* A1298C**	Egyptian (our study)	105	80	AA	46–19			Susceptible	
				AC	35–51			Protective	
	Jewish	93	377	CC	23–48			Susceptible	[29]
	Italian	217	251	CC	26–12			Susceptible	[5]
	African-American	138	53					No association	[7]
	Caucasian	393	50					No association	[7]
***TGFβ1* T869C**	Egyptian (our study)	105	80	TC	69–32			Susceptible	
				CC	18–29			Protective	
	Japanese (Nagoya)	155	110			T	160–101	Susceptible	[31]
	Japanese (Tsukuba)	137	225					No association	[10]
	Chinese	76	100	CC	11–33	C	63–113	Protective	[33]
	Chinese	105	100	CC	17–34	C	86–114	Protective	[34]
	Egyptian (Zagazig)	160	168			T	215–200	Susceptible	[32]
	Turkish	131	133					No association	[35]
	Caucasian (New Zealand)	117	140					No association	[36]
	Caucasian (UK)	395	401					No association	[37]
	Korean	143	148					No association	[38]
**ApaI**	Egyptian (our study)	105	80	aa (TT)	20–4			Susceptible	
	Spanish	120	200					No association	[51]
	Egyptian (Mansoura)	128	150	aa (TT)	26–10			Susceptible	[57]
	Tunisian	106	153					No association	[60]
**BsmI**	Egyptian (our study)	105	80	bb (GG)	48–19			Susceptible	
				BB (AA)	10–20	B (A)	57–81	Protective	
	Spanish	120	200					No association	[51]
	Korean	157	211					No association	[52]
	French	96	96					No association	[53]
	Hungarian	64	40					No association	[54]
	Turkish	98	122					No association	[55]
	Tunisian	108	152					No association	[56]
	Egyptian (Mansoura)	128	150	BB (AA)	13–36	B (A)	78–146	Protective	[57]
				bb (GG)	63–40			Susceptible	
	Egyptian (Zagazig)	200	150					No association	[58]
	German	62	40					No association	[59]
**FokI**	Egyptian (our study)	105	80					No association	
	French	100	100	FF (CC)	45–30	F (C)	133–108	Susceptible	[53]
	Turkish	98	122					No association	[55]
	Tunisian	108	152	FF (CC)	49–46	F (C)	147–164	Susceptible	[56]
	Egyptian (Mansoura)	128	150					No association	[57]
	German	62	40					No association	[59]
	Canadian	448	704	Ff (CT)	243–308			Susceptible	[62]
	Indian	112	125					No association	[61]
**TaqI**	Egyptian (our study)	105	80	TT (TT)	48–21	T (T)	143–81	Susceptible	
				tt (CC)	10–20			Protective	
	Spanish	120	200					No association	[51]
	Korean	157	120					No association	[52]
	French	95	95					No association	[53]
	Turkish	98	122					No association	[55]
	Egyptian (Mansoura)	128	150	TT (TT)	64–39	T (T)	179–152	Susceptible	[57]
				tt (CC)	13–37			Protective	
	German	62	40					No association	[59]
	Tunisian	106	153					No association	[60]
***TNFB* A252G**	Egyptian (our study)	105	80	AA	57–22	A	156–87	Susceptible	
				AG, GG	42–43, 6–15			Protective	
	Belgian	77	58					No association	
	Latvian (juvenile RA)	128	114					No association	
	Spanish	60	102					No association	
	Japanese	60	103					No association	
	Caucasian (UK)	388	399	GG	77–47	G	337–283	Susceptible	
	Portuguese (White)	388	269	AA	197–117	A	547–346	Susceptible	
	Saudi Arabian	106	126	AA, GG	32–15, 27–18			Susceptible	
				AG	47–93			Protective	
	Tunisian	108	226			G	84–131	Susceptible	
				AA	37–115			Protective	

The *MTHFR* A1298C was verified as a biomarker for RA disease in Jewish and Italian populations [5, 29]. The current results in this article supported the association of A1298C with RA susceptibility. This agreement may be due to these studied populations represent Mediterranean populations. Contradictory results were shown in American population with Caucasian and African ethnicities [7]. The negative findings found in the Americans might be due to the enrichment of the flour products in the US with folic acid since 1998 [1, 5]. The allele (1298C) was found to exhibit lower MTHFR enzyme activity, hyperhomocysteinemia, and decreased folate levels.

The *MTHFR* C677T was highly suggested for association with RA cases in Italian population [5]. This study demonstrated the association of *MTHFR* C677T with RA susceptibility in Egyptian population which had been addressed in Turkish population [30]. These similarities might be explained as Egypt and Turkey are Middle Eastern countries.

There were controversial results of *TGFβ1* T869C for the susceptibility to RA disease in different populations. The association between T869C variant and RA was confirmed in Japanese (Nagoya), Chinese and Egyptian populations [31–34]. Other results did not show association between RA and T869C in Caucasian (New Zealand and UK), Turkish, Japanese (Tsukuba) and Korean populations [10, 35–38]. Chang et al. conducted a meta-analysis on seven studies and resulted in contradictory outcomes. They concluded that T869C was associated with RA in Asian patients but not in non-Asian patients [39]. This conclusion was confirmed by Zhang et al. [40]. The (TT) genotype represented a risk factor for RA while (CC) genotype or (C) allele seemed to be protective to RA through a meta-analysis conducted by Zhou et al. [41]. *TGFβ1* results differed in our study and the study of [32] for the Egyptian population. This variation in results might be due to the sample sizes (our study: cases 105, controls 80 while Hussein et al.: cases 160, controls 168) or the age of patients (our study: 42.71 ± 12.07 years while Hussein et al.: 47.3 ± 9.3 years) or the disease duration (our study: 5.72 ± 4.35 years while Hussein et al.: 10.23 ± 7.5 years).

The results of the association between RA susceptibility and *TNFB* A252G have proven conflicting in different populations. By analyzing the possible influence of A252G on the susceptibility of RA in Belgian, Japanese, Latvian (juvenile RA), and Spanish populations, the results did not show any significant association [42–45]. The association between A252G variant and RA was confirmed in Caucasian (UK), white Portuguese, Saudi Arabian, and Tunisian populations [13, 46–48]. The current results for *TNFB* were consistent with the findings in Portuguese (White).

*HLA-DRB1* alleles were associated with RA susceptibility in the Egyptian population [49]. *TNFB* and *HLA-DRB1* are in LD, as they are located in the major histocompatibility complex (MHC) region (about 1000 kb apart from each other) [50]. So, the association between *TNFB* and RA susceptibility in the Egyptian population might be due to the LD between *TNFB* and *HLA-DRB1*.

The *VDR* polymorphisms and RA susceptibility showed unclear relations. There were no associations between *VDR* SNPs and RA in Spanish (BsmI, ApaI, TaqI), Korean (BsmI, TaqI), French (BsmI, TaqI), Hungarian (BsmI), Turkish (BsmI, TaqI, FokI), Tunisian (ApaI, BsmI, TaqI), Egyptian (Mansoura) (FokI), Egyptian (Zagazig) (BsmI), Indian (FokI), and German (BsmI, TaqI, and FokI) populations [51–61]. Other results showed association between *VDR* polymorphisms and RA susceptibility in French (FokI), Tunisian (FokI), Egyptian (Mansoura) (BsmI, TaqI, ApaI), and Canadian (North American Natives) (FokI) populations [53, 56, 57, 62].

For *VDR* gene polymorphisms, our results confirmed the previous results of Mosaad et al. [57]. These two studies supported the confirmation of the major role of (BsmI, TaqI, and ApaI) polymorphisms in RA susceptibility in the Egyptian population. BsmI results differed in our study and the study of [58] for the Egyptian population. This variation may be due to the studied gender (our study: both females & males, Hussein et al.: female only), the sample sizes (our study: cases 105, controls 80 while Hussein et al.: cases 200, controls 150) or the age of patients (our study: 42.71 ± 12.07 years while Hussein et al.: 57.3 ± 3.9 years) or the disease duration (our study: 5.72 ± 4.35 years while Hussein et al.: 1.25 ± 0.78 years).

## Conclusions

Direct associations between *TNFB*, BsmI, TaqI, *MTHFR* (C677T, A1298C), *TGFβ1*, and ApaI polymorphisms and RA susceptibility have been demonstrated in this study. In addition to the absence of a confirmed direct functional effect of FokI polymorphism, our results indicate that FokI have no role in RA susceptibility indirectly through the poor r^2^ values between FokI and all the *VDR* SNPs. Further studies with extended sample sizes (from the same population) are necessary to overcome the lack in power results and confirm our results in the Egyptian population. In addition, further investigations of other polymorphisms and its association with RA susceptibility may be helpful to clarify the pathogenesis of the disease.

## Supporting Information

S1 Table RFLP Conditions for the Identification of Polymorphisms Genotyped in the VDR Gene. (TIF)

S2 Table Power Results for the Studied SNPs. The table was generated using the *Genetic Power Calculator* program.
(TIF)
