# Supplementary figures and images for: Genetic Case-Control Study for Eight Polymorphisms Associated with Rheumatoid Arthritis
Source: PLoS One. 2015 Jul 6;10(7):e0131960. doi: 10.1371/journal.pone.0131960 (PMC4492599; doi:10.1371/journal.pone.0131960)

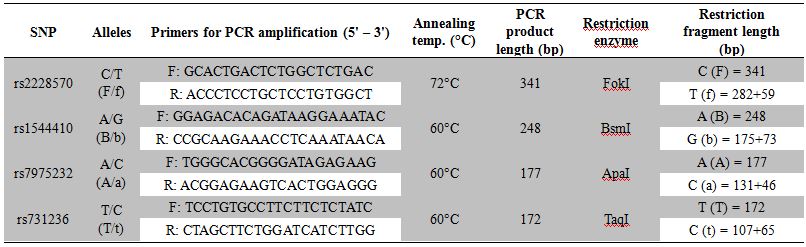

Supplement: S1 Table — (TIF) [file pone.0131960.s001.tif]

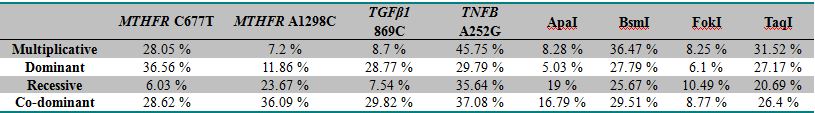

Supplement: S2 Table — The table was generated using the Genetic Power Calculator program. (TIF) [file pone.0131960.s002.tif]
